# Transtympanic Electrocochleography for the Diagnosis of Ménière's Disease

**DOI:** 10.1155/2012/852714

**Published:** 2012-01-26

**Authors:** Jeremy Hornibrook, Catherine Kalin, Emily Lin, Greg A. O'Beirne, John Gourley

**Affiliations:** ^1^Department of Otolaryngology and Audiology, Christchurch Hospital, 2 Riccarton Avenue, Christchurch 8011, New Zealand; ^2^Department of Communication Disorders, University of Canterbury, Christchurch 8020, New Zealand

## Abstract

This paper evaluated the diagnostic power of electrocochleography (ECochG) in detecting Ménière's disease (MD) as compared with two subjective assessment methods, including the clinical guidelines provided by the American Academy of Otolaryngology—Head and Neck Surgery Committee on Hearing Equilibrium and the Gibson score. A retrospective study of 250 suspected MD cases was conducted. The agreement between the three assessment methods was found to be relatively high, with a total reliability being higher than 70%. Participants who tested “positive” with ECochG exhibited a higher occurrence rate of asymmetric hearing threshold as well as the four MD symptoms, namely, vertigo, hearing loss, tinnitus, and aural fullness. The “positive” ECochG group also showed a high correlation between the ECochG measures in response to stimuli at adjacent frequency ranges, suggesting that the interfrequency ECochG correspondence may be sensitive to the presence of endolymphatic hydrops and thus may serve as a useful diagnostic marker for MD.

## 1. Introduction

Ménière's disease is an idiopathic inner-ear disorder [1, 2]. It is characterised by episodes of vertigo, roaring tinnitus, fluctuating sensorineural hearing loss, and a sense of aural fullness in the affected ear, with a combination of these signs or symptoms fluctuating over months and years [3]. The diagnosis of MD is normally made using the clinical guidelines set by the American Academy of Otolaryngology—Head and Neck Surgery Committee on Hearing and Equilibrium (AAO-HNS CHE) based on a selection of signs and symptoms [4]. The diagnosis of MD can also be assisted with measurements such as the Gibson score [5], electrocochleography (ECochG), and, more recently, magnetic resonance imaging [6, 7]. However, as the histological information and thus the confirmation of an MD diagnosis can only be obtained through postmortem biopsies [8], the administration of appropriate clinical diagnostic tools and treatment remains a challenging task. To facilitate the application of an instrumental, objective, and thus possibly more reliable approach in the diagnosis of an MD, this study investigated the agreement on the diagnosis of MD between ECochG and the two commonly used subjective methods, the AAO-HNS CHE guidelines and the Gibson score.

The AAO-HNS CHE guidelines for the diagnosis of MD were first established in 1972 and later revised in 1985 and 1995. The most recent version of the AAO-HNS CHE guidelines, as shown in Table 1, classifies the diagnosis of MD into four levels: “possible,” “probable,” “definite,” and “certain” [4]. It has been shown in a review study that 79.9% of the papers reviewed reported the use of the AAO-HNS CHE criteria; however, only 50% of those publications used the AAO-HNS CHE criteria correctly to diagnose MD [9]. As data may be less comparable between studies if the AAO-HNS CHE criteria are not strictly adhered to [10], there are concerns about the accuracy of the diagnosis made based on the AAO-HNS CHE criteria for some patients. Questions have been also raised regarding the limitations of the 1995 AAO-HNS CHE guidelines in its scope of symptom investigation, such as the length of vertigo attacks and the severity of tinnitus and aural fullness [11], and in its diagnostic power in detecting MD as compared with an approach based on Prosper Ménière's original description of the disease [10].

The Gibson score is a point system developed in 1991 by William Gibson [5]. It simplifies the diagnosis of MD through evaluating the interaction and dependence of the four typical components present in those with MD: vertigo, hearing, tinnitus, and aural fullness. The score is designed to be used with the clinical history of the patient. As shown in Table 2, each of these four components includes two (for tinnitus and aural fullness) or three (for vertigo and hearing) descriptions of the symptom. When a description applies to a patient, a point is given. A total score of 7 or higher indicates a diagnosis of MD [5]. This point system is considered a helpful clinical tool as it summarises the patient's clinical history with a quantification scheme assessing the extent of the four main MD symptoms in relation to one another. However, as the Gibson score is obtained based on subjective assessment, the diagnostic power of this point system also relies heavily on the accuracy of the information used to derive the score.

The problems associated with the use of a subjective assessment method in the diagnosis of MD arise mainly from its reliance on the signs and symptoms nonexclusive to the disease without a direct observation of the physiological changes related to the aetiology of the disease. The aetiology of MD has been linked to endolymphatic hydrops, with evidence from histological studies [12]. Endolymphatic hydrops refers to the swelling of the scala media from excessive accumulation of endolymph [13]. The audiometric testing approach is not sensitive or specific enough for an early detection of endolymphatic hydrops, which has been considered a consistent feature of MD and thus the primary pathology of MD [12].

Electrocochleography, which measures the auditory evoked response (AER) from the cochlea, provides a direct assessment of inner-ear function. An AER is an electrical response from the auditory system elicited by an acoustic stimulus [13], such as a click or a tone burst. With the ECochG method, the activity of the cochlea and VIIIth cranial nerve in response to acoustic stimuli can be monitored with an electrode either just outside, on, or through the tympanic membrane (called extratympanic, tympanic, and transtympanic ECochG, resp.). A ground electrode can be placed nearby, such as on the forehead, while two electrodes can be placed on the earlobes to obtain a differential recording [13]. Although ECochG is more invasive than other AERs, such as the auditory brainstem response (ABR), it has been documented in the literature for over 30 years as a tool for the diagnosis, assessment, and monitoring of patients with MD [14].

The ECochG waveform arises within the first two or three milliseconds after a rapid-onset acoustic stimulus. Originating from the cochlea and the eighth cranial nerve, the normal AER consists of three components: the cochlear microphonic (CM), summating potential (SP), and compound action potential (AP). Both CM and SP signals are generated by the hair cells of the cochlea in response to an acoustic stimulus [15, 16]. The CM potential, which is present throughout the whole duration of an acoustic stimulus, is the extracellular analogue of the alternating (AC) receptor current through the outer hair cells of the cochlea [17]. The CM reflects the instantaneous displacement of the basilar membrane and hair cell stereocilia in response to an acoustic stimulus and thus resembles a distorted version of the stimulus waveform [15, 16, 18]. Under normal conditions, the SP arises from the asymmetric transfer function of the inner hair cells, and is a direct current (DC) response [13, 15]. The AP is produced by fibres within the distal (cochlear) portion of the eighth cranial (auditory) nerve [13]. The compound AP signal represents a collective response resulting from numerous auditory nerve fibres firing synchronously [16]. The AP is usually larger than the SP, with a latency of approximately 1.5 ms [13]. Measurements commonly made on the ECochG waveforms are the amplitudes of the SP and AP components, using either a peak-to-trough or a baseline reference demarcation method [15].

Endolymphatic hydrops may change the ECochG waveforms by increasing the magnitude of the SP in response to clicks and tone bursts, creating an abnormally large potential [18–20]. The effect of endolymphatic hydrops on ECochG measures has been demonstrated [21]. With endolymphatic hydrops, the displacement of the basilar membrane towards scala tympani moves the outer hair cell (OHC) operating point closer to that of the inner hair cells (IHCs), resulting in an increase of DC component in the OHC receptor current, adding the OHC SP to the IHC SP. Furthermore, as there are three times as many OHCs as IHCs, the SP magnitude is greatly increased. In contrast, the amplitude of the compound AP is decreased due to an OHC motor loss leading to reduced efficiency of the electromechanical transduction in the MET channels. Asai and Mori [22] tested eight patients with MD using the ECochG method and reported that the amplitude of AP decreased with an increase of hearing threshold at high frequencies (2–8 kHz) but altered independently of the hearing threshold at low frequencies (0.25–1 kHz) while the SP remained constant throughout the fluctuation of hearing loss. An SP-to-AP ratio in response to a click stimulus with a value greater than 40–50% has also been shown to indicate the presence of endolymphatic hydrops [23]. The amplitudes of the SP and AP (measured in microvolts) have long been used to determine if a person has normal hearing, sensorineural hearing loss, retrocochlear hearing loss, or MD [13]. As small changes in the endolymph fluid of the cochlea can affect the ECochG waveforms, the ECochG appears to be sensitive to the presence of MD.

 A misdiagnosis of MD will result in not only wastage and inefficiency in the use of medical resources but also a traumatic and irreversible impact on the patient. As the AAO-HNS CHE criteria approach relies on self-report of the symptoms, the classification of the clinical diagnosis remains vague and subjective. An objective, instrumental method is needed to improve the diagnosis and management of MD. Conlon and Gibson [20] have shown in a study of 2,964 ears that ECochG achieved a higher level of accuracy in detecting MD than conventional clinical examination. However, more empirical evidence from independent studies evaluating the diagnostic power of ECochG is needed to facilitate the clinical application of ECochG as a clinical tool and enhance the understanding of the underlying pathophysiology of MD. This study aims to gauge the intermethod reliability in the detection of MD between the ECochG method and two established subjective methods, as well as comparing participants who tested “positive” with the ECochG measures and those who tested “negative” to explore how measurement variability may reflect the underlying pathophysiology.

## 2. Method

### 2.1. Participants and Participant's Task

Medical records, including results from the participant's hearing test, clinical examination, and ECochG recordings, were retrieved from a hospital database. The participants had been referred to the Department of Otolaryngology at Christchurch Public Hospital (Christchurch, New Zealand) in the period from year 1994 to 2009 for the diagnosis of MD, had given informed consent to data collection for the research, and had complete records of the results from the assessment made with the AAO-HNS CHE criteria, Gibson score, and ECochG testing. Ethical approvals were obtained from the New Zealand Ministry of Health, Health and Disability Ethics Committees, and the University of Canterbury Ethics Committee. Based on a quota sampling strategy, a total of 250 participants (117 females and 133 males) were included. Ethnicity data is routinely collected in New Zealand. Participants included mostly individuals of European descent (94.4%), but there were also six Asians, three Māori, one Pacific Islander, and four from other ethnic groups. The age of the participants ranged from 9 to 88 years (mean = 53 years, SD = 14.42).

### 2.2. Instrumentation

**Figure 1:**
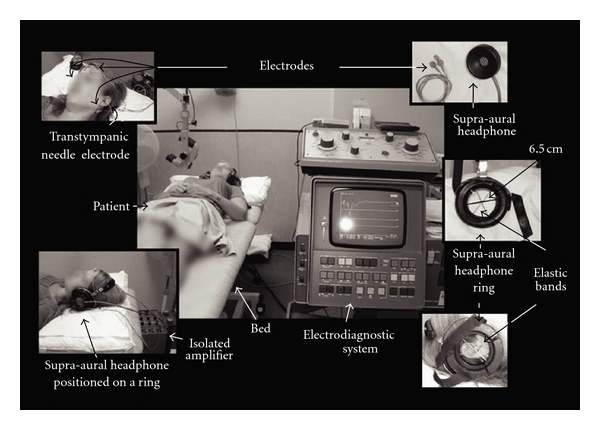
Instrumentation setup.

Instruments used for the recording of ECochG signals included an electrodiagnostic system (Amplaid MK 15, Milan, Italy), consisting of disposable electrodes (Ambu Blue Sensor electrodes, Denmark), a sterilised transtympanic (TT) needle electrode, phenol, elastic bands, and a supra-aural headphone. The TT ECochG method, which involves a recording needle electrode being placed down the ear canal and through the tympanic membrane to rest on the promontory of the cochlea, was used. As compared to the extratympanic ECochG method, which involves an electrode resting on the tympanic membrane or against the skin of the external auditory meatus [16, 24], the TT ECochG method has the advantage of having the recording electrode at a close proximity to the cochlea. This advantage enables large ECochG response waveforms with a minimal signal averaging required [16, 25] resulting in a more reliable and reproducible output signal [24]. As shown in Figure 1, the four electrodes were placed on the participant's head, with one with an attachment to a TT needle, one on the forehead, and one on each of the two earlobes. The four electrodes were connected through wires to an isolated biological amplifier, which was connected to the electrodiagnostic system. The supra-aural headphones (TDH39) were also connected to the electrodiagnostic system.

### 2.3. Procedure

During the initial visit, a hearing test and a clinical examination were conducted. For the hearing test, an audiologist completed a diagnostic air conduction pure tone audiogram using the modified Hughson-Westlake procedure to detect the thresholds at 0.25, 0.5, 1, 2, 4, and 8 kHz for each subject bilaterally. The bone conduction threshold was obtained at the frequencies where the air conduction threshold was greater than 20 dB HL to determine whether a conductive or sensorineural hearing loss is present. The ECochG recording was conducted approximately two months after the initial clinical examination with the otolaryngologist.

**ECochG Recording**

For ECochG recording, the skin was prepared with Medi-Swab alcohol skin cleansing swabs (BSN Medical) before the ground electrode was placed on the forehead and an active electrode attached to each two earlobe. The tympanic membrane was anaesthetised with a drop of phenol before the insertion of a sterilised TT needle electrode piercing through the tympanic membrane to rest in the round window niche. Elastic bands attached to a 6.5 cm diameter ring were positioned over the auricle of the test ear to secure the needle in place. The participant was instructed to lie in a supine position during testing to decrease muscle noise while the recording took place. A headphone sound source was located on the ring over the test ear.

The tone bursts used to elicit the ECochG AER were at 0.5, 1, 2, and 4 kHz, respectively. The intensity of the tone bursts was 100 dB nHL for the frequency at 4 kHz and 90 dB nHL for the frequencies at 0.5, 1, and 2 kHz. The rise and fall time specified for the tone burst was 1 ms, with a 14 ms plateau, a total duration of 16 ms. The repetition rate of the tone bursts was 30.1 per second. The 100 *μ*s clicks were presented at an intensity of 90 dB nHL, with an alternating polarity at a rate of 10 times per second. A total of 1,024 tone bursts per run were delivered and, along with the response signals, recorded with an analysis time window of 30 ms. A total of 256 clicks per run were delivered and, along with the response signals, recorded with an analysis time window of 10 ms. Both the acoustic stimuli and the AER signals were filtered respectively through a band-pass filter, which consisted of a low-pass filter at 3 kHz with a 12 dB per octave filter slope and a high-pass filter at 0.5 Hz with a 6 dB per octave filter slope.

### 2.4. Measurements

**Figure 2:**
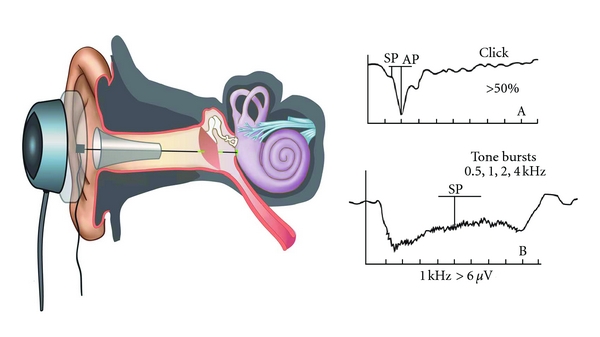
A schematic illustration of the placement of the transtympanic needle electrode and the electrocochleographic waveforms.

Data retrieved from the medical records regarding the results of the pure tone air conduction audiogram were hearing thresholds measured at five frequencies: 0.5, 1, 2, 4, and 8 kHz. A mean threshold, termed a pure tone average (PTA), was calculated for each ear tested by averaging the thresholds measured at 0.5, 1, and 2 kHz for each ear. The components relevant to the AAO-HNS CHE criteria as shown in Table 1 were noted by an otolaryngologist for each participant. Any unique details of the participant's history were also noted, along with the type of hearing loss and the presence of vertigo attacks, tinnitus, aural fullness, and disequilibrium. A Gibson score was also calculated by an otolaryngologist for each participant. The scoring was based on the standard point system as shown in Table 2. Measurements from the ECochG signals recorded for each participant included measures derived from signals in response to tone bursts and clicks, respectively. The SP amplitude (in *μ*V), AP amplitude (in *μ*V), and an SP-to-AP amplitude ratio (in %) were obtained for each ear for tone bursts generated at 0.5, 1, 2, and 4 kHz, respectively, and for clicks.  Figure 2 illustrates the measures derived from the ECochG waveforms as well as the placement of the TT needle electrode used to obtain the ECochG signals.

### 2.5. Data Analysis

The participant's basic demographic information, including name, date of birth, sex, and identity number, and the specific test results for auditory thresholds, Gibson score, ECochG measurement, and components related to the AAO-HNS CHE criteria were extracted from the medical records for each participant. Based on the symptoms indicated on the participant's medical records, the 1995 AAO-HNS CHE criteria as previously described (see Table 1) were used to determine whether a participant would be classified as having “possible,” “probable,” or “definite” MD. Each Gibson score was obtained based on the scores recorded for the components included in the point system [26]. Based on the ECochG measures, participants with any recorded SP values for clicks or tone bursts greater than the normative data values, as specified in Table 3 [27], were classified as “positive” and those with none of the recorded SP values greater than the normative data values as “negative.”

### 2.6. Statistical Analysis

Measures yielded by the three diagnostic tools, including ECochG, AAO-HNS CHE, and Gibson score, were compared. With the ECochG diagnostic test taken as the hypothetical “gold standard,” the sensitivity and specificity of the two subjective tests, including AAO-HNS CHE criteria and Gibson score, were calculated (see Table 4). An ROC curve was plotted for both subjective tests. The best cutoff point for each of the two ROC curves was chosen to assess intermethod reliability. To determine the level of agreement between the three diagnostic tools, four types of intermethod reliability, including total, point-by-point, occurrence, and nonoccurrence reliability, were calculated based on the formula as shown in Table 5. With “positive” and “negative” identifications made through the three diagnostic tools respectively, a series of chi-square tests were conducted to compare the number of participants in groups related to different classifications. A series of correlation procedures were also conducted to determine the relationships between a selection of ECochG measures in the “positive” and “negative” groups, respectively. The significance level was set at 0.1, with adjustments using the Bonferroni correction for multiple testing.

## 3. Results

**Figure 3:**
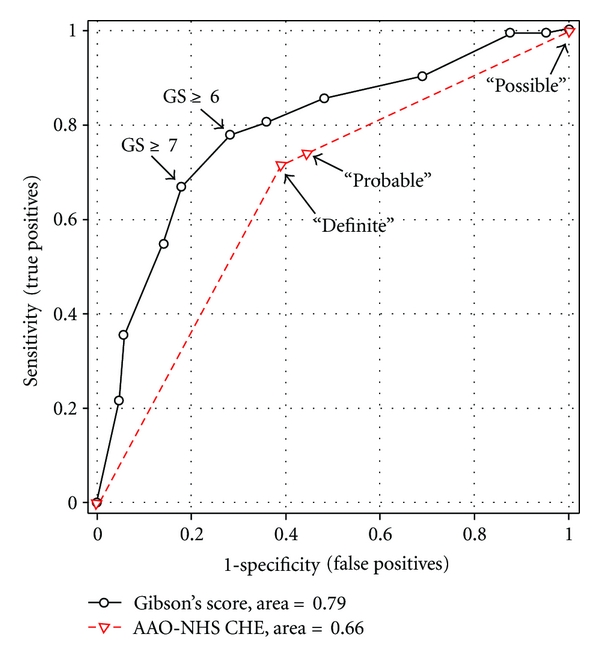
A receiver operating characteristic (ROC) curve of the Gibson score test showing 11 cutoff points (right to left from 0 to 10) and that of the AAO-HNS CHE test showing 3 cutoff points (“possible,” “probable,” and “definite”).

The ROC curves for the two subjective tests, with the ECochG diagnosis taken as the hypothetical “gold standard,” were plotted in Figure 3. Three cutoff points (“possible,” “probable,” and “definite”) were marked on the ROC curve for the AAO-HNS CHE criteria and eleven cutoff points (from 0 to 10 in steps of one) on that for the Gibson score. The area under the curve for the Gibson score was found to be significantly greater than that for the AAO-HNS CHE criteria (chi-square = 22.51, df = 1, *p* < 0.001), indicating that the Gibson score was more powerful in discriminating between “positive” and “negative” ECochG cases. The ROC curve for the Gibson score appears to have a sharp turn at the cutoff point of seven, which falls on the cutoff value recommended by Gibson [27] to make a positive MD diagnosis. The shape of the ROC curve for the Gibson score is typical of an ROC curve constructed when a real “gold standard” diagnosis is available for comparison, suggesting that the ECochG method provides a valid alternative to the two subjective methods.

**Figure 4:**
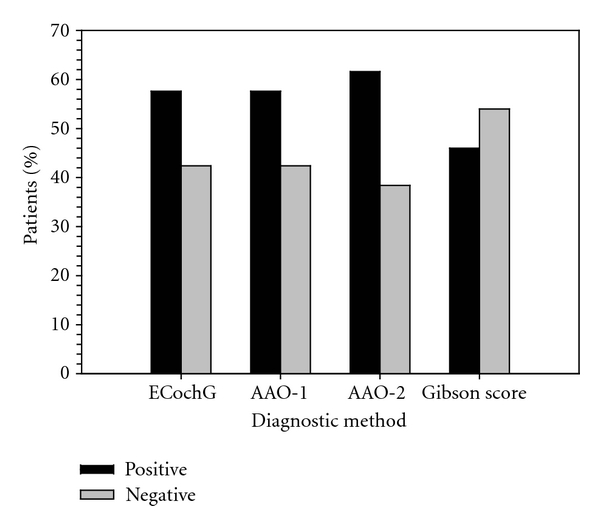
Percentage of participants identified as “positive” and “negative,” respectively, using different diagnostic methods, including ECochG method, AAO-HNS CHE criteria with only “definite” being classified as “positive” (AAO-1), AAO-HNS CHE criteria with both “definite” and “probable” being classified as “positive” (AAO-2), and Gibson score with 7 as the cutoff point.

**Figure 5:**
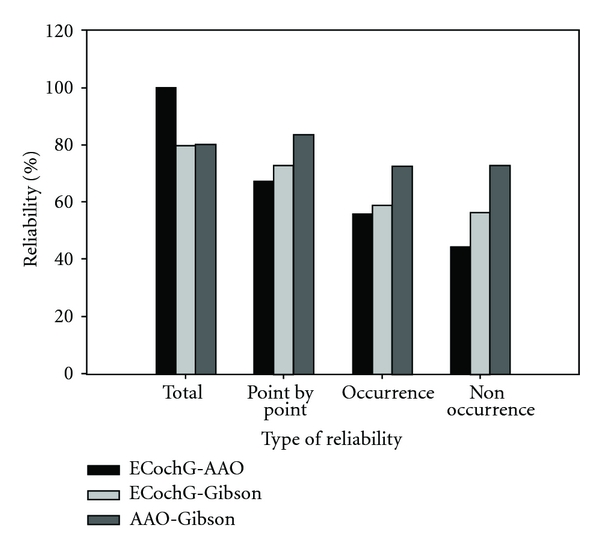
Total, point-by-point, occurrence, and nonoccurrence reliability between ECochG method and AAO-HNS CHE criteria, between ECochG method and Gibson score, and between AAO-HNS CHE criteria and Gibson score.

Figure 4 shows the percentage of participants in the “positive” and “negative” groups as classified with different diagnostic methods. The AAO-HNS CHE criteria were changed to a dichotomous classification in two ways, one with “definite” and “probable” identified as “positive” (“AAO-definite/probable”) and the other with only “definite” identified as “positive” (“AAO-definite”). The Gibson score with a cutoff point of 7 was used for making the diagnosis. As shown in Figure 4, both ECochG and AAO-HNS CHE methods identified more positive cases than negative cases while the approach with the Gibson score identified more negative cases than positive cases.  Figure 5 illustrates results for a series of intermethod reliability measures between ECochG and AAO-HNS CHE criteria, between ECochG and Gibson score, and between AAO-HNS CHE criteria and Gibson score. As shown in Figure 5, the point-by-point, occurrence, and nonoccurrence reliability were highest between AAO-HNS CHE criteria and Gibson score and the total reliability was highest between ECochG and AAO-HNS CHE criteria. There were a greater proportion of males (66%) identified as “positive” ECochG than females (43%). Based on the AAO-HNS CHE criteria, the majority of the participants diagnosed as “definite MD” were between 51–60 years (18.8%), those as “probable MD” were between 41 and 50 years (1.34%), and those as “possible MD” were between 51 and 60 years (6.71%). The majority of the participants who tested “positive” for MD with ECochG were between the ages of 41 and 80 years of age (87.24%). For the “positive” ECochG cases, a significantly higher amount of participants had unilateral (left ear: 36.9%; right ear: 45%) than bilateral MD (18.1%). Furthermore, more bilateral MD cases were identified using the ECochG method (27 cases) as compared with the Gibson score (1 case). A comparison of the tone burst and click ECochG results for the total number of participants who tested as “positive” with ECochG revealed that the tone burst method (59.5%) generally identified more “positive” cases than the click method (24.8%). The ECoChG classification results based on the composite click and tone criteria as shown in Table 3 were found to be most highly correlated with the results obtained based on measures with tone bursts at 1 kHz alone (*r* = 0.79), followed in order by those with tone bursts at 2 kHz alone (*r* = 0.62), tone bursts at 0.5 kHz alone (*r* = 0.45), tone bursts at 4 kHz alone (*r* = 0.43), and clicks alone (*r* = 0.42).

### 3.1. Distributions of MD Symptoms

**Figure 6:**
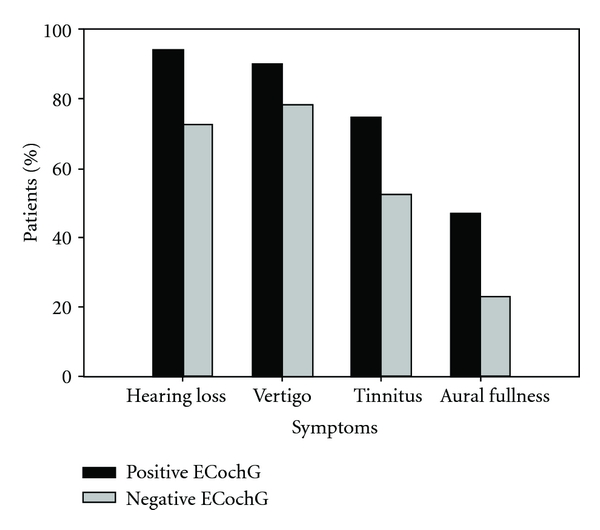
Percentage of patients showing each of the four key symptoms of MD.

Figure 6 shows a comparison between “positive” and “negative” ECochG cases on the prevalence of the four key MD symptoms, namely, hearing loss, vertigo, tinnitus, and feeling of aural fullness. As shown in Figure 6, participants who tested “positive” with ECochG exhibited a higher occurrence rate of the four symptoms than those who tested “negative.” Both hearing loss and vertigo were the most prevalent symptoms, followed in order by tinnitus and feeling of aural fullness (see Figure 6). No significant difference on the occurrence rate was found between hearing loss and vertigo (“positive” ECochG: chi-square = 0.098, df = 1, *p* = 0.75; “negative” ECochG: chi-square = 0.615, df = 1, *p* = 0.43). A significant difference on the occurrence rate was found for all the other pairwise comparisons, including the comparison between hearing loss and tinnitus (“positive” ECochG: chi-square = 44.5, df = 1, *p* < 0.001; “negative” ECochG: chi-square = 8.621, df = 1, *p* = 0.003), hearing loss and aural fullness (“positive” ECochG: chi-square = 74.37, df = 1, *p* < 0.001; “negative” ECochG: chi-square = 51.65, df = 1, *p* < 0.001), vertigo and tinnitus (“positive” ECochG: chi-square = 7.645, df = 1, *p* = 0.006; “negative” ECochG: chi-square = 14.73, df = 1, *p* < 0.001), vertigo and aural fullness (“positive” ECochG: chi-square = 23.69, df = 1, *p* < 0.001; “negative” ECochG: chi-square = 63.88, df = 1, *p* < 0.001), and tinnitus and aural fullness (“positive” ECochG: chi-square = 6.07, df = 1, *p* = 0.014; “negative” ECochG: chi-square = 18.79, df = 1, *p* < 0.001).

### 3.2. Hearing Loss Patterns

**Figure 7:**
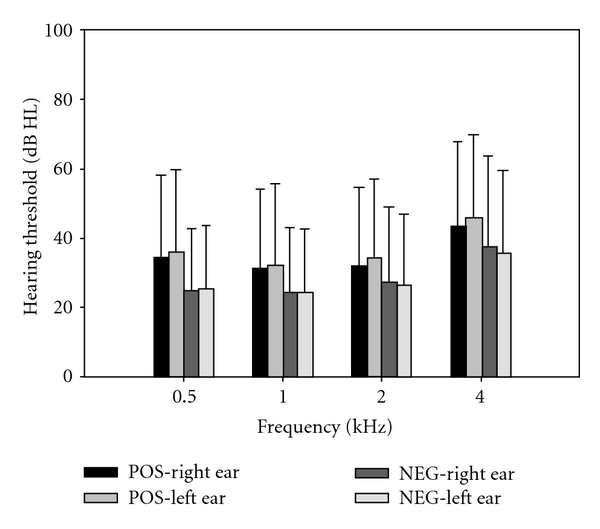
Means and standard deviations of the hearing thresholds as measured at 0.5, 1, 2, and 4 kHz for the “positive” and “negative” ECochG groups.

Regardless of the ECochG diagnosis, there was generally a higher proportion of asymmetrical hearing thresholds (“positive” ECochG: 88.7%; “negative” ECochG: 72.5%) than symmetrical hearing thresholds. The “positive” ECochG group was found to have a significantly higher proportion of participants showing asymmetrical hearing thresholds than the “negative” ECochG group (chi-square = 9.66, df = 1, *p* = 0.002). While the occurrence rates across the three types of between-ear contrast were not significantly different in the “negative” ECochG group (chi-square = 2.51, df = 2, *p* = 0.286), they were significantly different in the “positive” ECochG group (chi-square = 46.15, df = 2, *p* < 0.001), with a significantly higher occurrence rate of asymmetrical threshold (“left ear poorer”: 43.3%; “right ear poorer”: 45.4%) as compared with symmetrical threshold shift (11.3%), whether the poorer threshold was found in the left ear (chi-square = 34.59, df = 1, *p* < 0.001) or the right ear (chi-square = 38.548, df = 1, *p* < 0.001). For the “positive” ECochG group, the occurrence rates for the “left ear to be the poorer ear” or “right ear to be the poorer ear” were not significantly different (chi-square = 0.058, df = 1, *p* = 0.811). As for the level of between-ear threshold difference, a comparison between the “positive” and “negative” ECochG groups on the proportions of participants with low (0–15 dB HL) and high (equal or greater than 20 dB HL) between-ear threshold differences revealed that a significantly higher proportion (60.3%) of “positive” ECochG cases exhibited a “high” between-ear threshold difference (chi-square = 11.12, df = 1, *p* < 0.001) while a significantly lower proportion (18.3%) of “negative” cases showed a “high” between-ear threshold difference (chi-square = 84.84, df = 1, *p* < 0.001).  Figure 7 shows the average hearing thresholds at 0.5, 1, 2, and 4 kHz for the “positive” and “negative” ECochG groups, respectively. An inspection of Figure 7 revealed that the “negative” ECochG group had a lower average hearing threshold at all frequencies than the “positive” ECochG group, with the highest hearing threshold being at 4 kHz for both “positive” and “negative” ECochG groups.

### 3.3. Relationships between ECochG Measures

Table 6 shows the results from a series of Pearson's Product Moment correlation procedures conducted to determine the relationships between the SP/AP ratios obtained from adjacent frequencies in the “positive” and “negative” groups as classified by the three diagnostic methods, respectively. With the ECochG diagnosis, SP/AP ratios measured at adjacent frequencies (i.e, 0.5 versus 1 kHz, 1 versus 2 kHz, and 2 versus 4 kHz) were moderately or highly correlated more often in the “positive” group than in the “negative” group. To further explore the interfrequency variation on the SP/AP measure, the coefficient of variation (COV) of the mean and standard deviation of the SP/AP ratios extracted from the four frequencies was obtained for each ear. The COV was defined as 100 times the ratio of standard deviation to mean. The value from the ear with a higher COV was selected for each participant for statistical analysis. Results from a two-way (2 diagnostic groups X 3 diagnostic methods) analysis of variance conducted on the COV measures revealed no significant method effect (F (2, 726) = 0.236, *p* = 0.79) but a significant diagnostic group effect (F (1, 726) = 26.244, *p* < 0.001) and a significant method by group interaction effect (F (2, 726) = 5.063, *p* = 0.007). Post hoc pairwise comparison tests revealed that the COV in the “positive” group (mean = 79.7) was significantly lower than that in the “negative” groups (mean = 115.4) only when the classification was based on the ECochG method.

## 4. Discussion

The purpose of this study was to evaluate how ECochG may function as a diagnostic tool for MD as compared with the conventional subjective methods. Both ECochG and AAO-HNS CHE criteria were found to independently diagnose a similar amount of participants with MD. The point-by-point intermethod reliability was found to be moderate among the three diagnostic methods. In the literature, the ECochG method has been found to be more specific but not adequately sensitive (20–65%) for the diagnosis of MD if the diagnosis was made based on cutoff values [14]. It has been shown, however, that the sensitivity of the ECochG test can be greatly improved (92%) if the amplitude and duration of the SP and AP are taken into consideration for setting the diagnostic criteria [14]. Therefore, the use of cutoff scores to obtain a diagnosis in this study may have contributed to some of the diagnostic disagreement with the other two diagnostic tools. The present finding that participants who tested “positive,” regardless of the diagnostic method used, showed a greater correspondence between the SP/AP ratios at adjacent frequencies (i.e., 0.5 versus 1 kHz, 1 versus 2 kHz, and 2 versus 4 kHz) than those who tested “negative” suggests that the correlations between these ECochG measures at adjacent frequencies may be useful for differentiating the impact of endolymphatic hydrops on the auditory system from that of other pathologies. In other words, the ECochG method may be more sensitive to the presence of MD if applied with a better discriminating rule, such as one that reflects the consistency of the measures between adjacent frequencies.

Another source of disagreement between the ECochG method and the other two subjective methods may be related to the fluctuating nature of the MD symptoms. In the early stage of MD, in particular, if the ECochG test is administered when no symptoms are present, it is unlikely that a positive diagnosis will be made. Due to the time it takes for an appointment which includes a hearing test, it is not practical to assess a person with ECochG at an initial consultation and there may be a long hospital waiting list for the test to be done. It is normally considered costly, time inefficient, and impractical for the participant, physician, and audiologist to repeat the ECochG test until a participant is fully symptomatic of MD. As a result, the participant may be in tremendous discomfort for years if they are waiting on a positive ECochG result. As the AAO-HNS-CHE criterion is a subjective diagnostic tool, the application of the criteria is susceptible to rating inconsistency. In addition to providing an objective and physiological measure of the pathology, the ECochG method, which assesses each ear independently, also has the advantage of taking unilateral and bilateral MD into diagnostic consideration. The high agreement between the ECochG method and the two subjective assessment methods suggests that ECochG used in combination with another assessment tool for the diagnosis of MD, such as the AAO-HNS CHE criteria or the Gibson score, would be beneficial. Therefore, repeated measures at different points of time or administering the ECochG test around the time when symptoms occur would be useful for enhancing the power of the ECochG method as a diagnostic tool.

It has been noted that clicks evoke a clear action potential due to their sharp onset but can cause acoustic ringing and distort the SP unpredictably. In contrast, tone bursts have a longer duration, which enhances the differentiation between SP and AP [28]. In an ECochG study of 42 normal ears, 48 ears with sensorineural hearing loss, and 80 Ménière's ears, the 1 kHz tone burst was shown to result in a higher accuracy level than the click in assisting in the clinical diagnosis of MD [28]. Likewise, tone bursts were found in an ECochG study of 2,421 ears to have the advantage over clicks in showing better frequency selectivity for monitoring the degree of endolymphatic hydrops at specific turns of the cochlea [25]. The present finding that the tone burst ECochG method yielded more “positive” ECochG cases than the click ECochG method agrees with the previous observation that a tone burst, especially at 1 kHz, is more sensitive than a click in eliciting AER for the diagnosis of MD.

There was a common observation in the literature that MD typically occurred unilaterally rather than bilaterally [29], with a reported estimate that bilateral MD occurred in one in three participants [30]. As bilateral MD is considered a natural progression for the disease in some people, it is of importance that every attempt at conservation of the nonsymptomatic ear should be made as early as possible. In addition, almost all participants with a “positive” ECochG diagnosis were found in this study to exhibit hearing loss and vertigo and to a lesser extent, tinnitus and aural fullness. Clinical guidelines suggest that a participant with a 20 dB HL or greater difference between audiometric thresholds should be referred to an otolaryngologist for evaluation of asymmetrical hearing thresholds. As many of the majority of participants with a large threshold difference between ears in this study were found to show a positive ECochG result and a “definite MD” AAO-HNS CHE diagnosis, asymmetrical hearing threshold, along with positive ECohG, may be a dominant early sign of MD.

The onset of MD is usually said to be around the fourth or fifth decade of life [4, 31]. In this study, most of the participants identified as “definite MD” are between the ages of 41 and 80, making this sample a much older population of MD patients. In addition, the present study showed that more males had MD than females. As the literature does not indicate that MD is more common in males [32], more investigations are needed regarding the gender difference in the prevalence of MD. Future studies extending to other regions, as well as involving the analysis of the progression of hearing loss in the MD participants, are needed.

## 5. Conclusions

The ECochG method was found to be in better agreement with the Gibson score than other diagnostic measures. The finding that there were still some disagreements among the three assessment methods in the diagnosis of MD suggested that a diagnosis of MD should not be made with a single diagnostic tool in their present forms of application. As there was some evidence suggesting that the signs of MD may be distinguishable from those of other causes based on the interfrequency relationship between ECochG measures obtained at adjacent frequencies, the consistency between ECochG measures across adjacent frequencies needs further investigation.

## Figures and Tables

**Table 1:** The 1995 AAO-HNS criteria for the diagnosis of Ménière's disease (adapted from [4, page 182]).

Category	Criteria
“Possible”	(i) Episodic vertigo of the Ménière type (i.e., spontaneous rotation vertigo lasting for 20 minutes or greater, often accompanied by disequilibrium which may last for days as well as nausea and rotatory nystagmus)
	(ii) Without documented hearing loss or with sensorineural hearing loss, fluctuating or fixed, with disequilibrium but without definitive episodes
	(iii) Other causes of vertigo excluded

“Probable”	(i) One definitive episode of vertigo
	(ii) Audiometrically documented hearing loss on at least one occasion
	(iii) Tinnitus or aural fullness in the treated ear
	(iv) Other causes excluded

“Definite”	(i) Two or more definitive spontaneous episodes of vertigo lasting for 20 minutes or longer
	(ii) Audiometrically documented hearing loss on at least one occasion
	(iii) Tinnitus or aural fullness in the treated ear
	(iv) Other causes of vertigo excluded

“Certain”	Definite Ménière's disease, plus postmortem histopathologic confirmation

**Table 2:** The point system of the Gibson score (adapted from[5, page 109]).

Parameter	Description	Points
Vertigo	(i) Rotational vertigo	1
	(ii) Attacks of rotational vertigo lasting over 10 min	1
	(iii) Rotational vertigo associated/linked with one or more of: hearing loss, tinnitus, or aural pressure	1

Hearing	(i) Sensorineural hearing loss	1
	(ii) Fluctuating sensorineural hearing loss	1
	(iii) Hearing loss or fluctuation associated/linked with one or more of vertigo, tinnitus, or aural pressure	1

Tinnitus	(i) Peripheral tinnitus lasting over 5 min	1
	(ii) Tinnitus fluctuating or changing with one or more of vertigo, hearing loss, or aural pressure	1

Aural pressure	(i) Constant aural pressure lasting over 5 min	1
	(ii) Aural pressure fluctuating or changing with one or more of vertigo, hearing loss, or tinnitus	1

Maximum score		**10**

**Table 3:** Electrocochleography criteria [27]. The diagnostic level was chosen as the nearest whole figure to the level which provides a false-positive diagnosis rate of 5%. The likelihood of hydrops is a Gibson score of >7/10 [6]. Clicks: abnormal if SP/AP ratio ≥0.50.

Tone bursts		
Tone burst frequency	Hearing level dBHL	Abnormal if SP ≤

0.5 kHz (75 dBHL)	Under 25	−2 *μ*V
	20–35	−2 *μ*V
	40–55	−2 *μ*V
	60–75	−1 *μ*V

1 kHz (90 dBHL)	Under 25	−6 *μ*V
	20–35	−6 *μ*V
	40–55	−6 *μ*V
	60–75	−3 *μ*V

2 kHz (100 dBHL)	Under 25	−9 *μ*V
	20–35	−7 *μ*V
	40–55	−5 *μ*V
	60–75	−5 *μ*V

4 kHz (75 dBHL)	Under 25	−9 *μ*V
	20–35	−5 *μ*V
	40–55	−5 *μ*V
	60–75	−5 *μ*V

**Table 4:** Formula for calculating the diagnostic power of the two subjective tests, respectively, as compared with the diagnosis based on ECochG measures.

		ECochG diagnosis	
		(hypothetical “gold standard”)	
		* Positive*	*Negative *
Results from AAO-HNS CHE (or Gibson score)	*Positive*	a (true positive)	b (false positive)
	*Negative*	c (false negative)	d (true negative)

Sensitivity = a/(a+c).

Specificity = d/(b+d).

Positive predictive value = a/(a+b).

Negative predictive value = d/(c+d).

**Table 5:** Conditions given for the calculation of four types of intermethod reliability (formula: reliability = (a/b) × 100).

Reliability	Conditions
Total	a = the smaller frequency of “positive” identification by one test
	b = the larger frequency of “positive” identification by the other test

Point by point	a = the number of cases with the same identification from both tests
	b = the total number of cases

Occurrence	a = the number of cases who tested “positive” in both tests
	b = the number of cases who tested “positive” at least in one test

Nonoccurrence	a = the number of cases who tested “negative” in both tests
	b = the number of cases who tested “negative” at least in one test

**Table 6:** Correlations (Pearson's r) between SP/AP ratios obtained at 0.5, 1, 2, and 4 kHz in the “positive” (POS) and “negative” (NEG) cases classified by three diagnostic methods.

		0.5 kHz		1 kHz		2 kHz	
		POS	NEG	POS	NEG	POS	NEG
*ECochG:*							
1 kHz	R	0.49	0.20				
	L	**0.52**	0.44				
2 kHz	R	0.37	0.22	**0.70**	**0.63**		
	L	0.37	0.38	**0.72**	0.47		
4 kHz	R	0.26	0.19	0.42	0.21	0.48	0.31
	L	0.22	0.23	0.35	**0.51**	**0.53**	0.34
*AAO-HNS CHE (“possible” as negative):*							
1 kHz	R	**0.50**	0.43				
	L	**0.61**	**0.50**				
2 kHz	R	0.42	0.36	**0.74**	**0.75**		
	L	0.45	**0.51**	**0.71**	**0.69**		
4 kHz	R	0.39	0.17	0.43	0.48	0.46	**0.57**
	L	0.21	0.41	0.29	**0.65**	**0.52**	0.47
*Gibson score (cutoff point at 7):*							
1 kHz	R	**0.51**	0.41				
	L	**0.60**	**0.51**				
2 kHz	R	0.46	0.31	**0.78**	**0.70**		
	L	0.41	**0.50**	**0.70**	**0.69**		
4 kHz	R	0.42	0.16	0.43	0.46	0.47	**0.54**
	L	0.17	0.43	0.22	**0.64**	0.44	**0.55**

*Significant correlations with a coefficient above 0.5 were in boldface.
